# Perioperative Cerebral Metabolism in Patients Under General Anaesthesia With Sevoflurane or Propofol: Evaluation of the Arterial and Jugular Bulb Metabolome

**DOI:** 10.1155/anrp/8559251

**Published:** 2026-10-23

**Authors:** Chenghong Wang, Sike Ma, Xudan Xing, Qian Liu, Yi Yang, Qian Lei, Juan Liao, Peng Li

**Affiliations:** ^1^ Department of Anesthesiology Sichuan Provincial People’s Hospital School of Medicine University of Electronic Science and Technology of China Chengdu China uestc.edu.cn; ^2^ Department of Anesthesiology Wenjiang Hospital of Sichuan Provincial People’s Hospital Chengdu Wenjiang District People’s Hospital Chengdu China; ^3^ Department of Stomatology Sichuan Provincial People’s Hospital School of Medicine University of Electronic Science and Technology of China Chengdu China uestc.edu.cn

## Abstract

**Objective:**

Arterial serum was generally used to uncover shifts in the metabolic profile induced by surgery and anaesthesia. The specific perioperative metabolic alterations in the brain remain largely unknown. We aimed to detect perioperative changes in cerebral metabolism by arteriovenous gradients of circulating metabolites across the brain using liquid chromatography–tandem mass spectrometry (LC‐MS) analysis.

**Methods:**

The prospective study was conducted at Sichuan Provincial People’s Hospital, China. Twenty patients with unilateral pulmonary lesion were recruited. Patients undergoing thoracic surgery were randomised to receive general anaesthesia with sevoflurane or propofol. Blood samples from radial artery and jugular bulb were collected before and after first anaesthetic administration, at the end of 60 min of maintenance of anaesthesia and 60 min after patients regained consciousness. LC‐MS‐based untargeted metabolomics strategies were used to determine the difference in abundance of circulating metabolites between artery and jugular bulb.

**Results:**

A total of 3900 metabolites were detected. Potential critical alterations of metabolites including phosphatidylcholine (PC), phosphatidylethanolamine (PE), choline, isocitrate and lactate and pathways were identified. Brain took up PC and PE in group sevoflurane but secreted these compounds in group propofol. Additionally, propofol was associated with the release of choline and isocitrate from brain. In both groups, lactic acid entered brain intraoperatively.

**Conclusion:**

This work may offer a comprehensive mapping of perioperative alterations of cerebral metabolism in patients under general anaesthesia and a new direction for further studies in pharmacology of general anaesthetics and pathogenesis of perioperative brain dysfunction.

**Trial Registration:** Chinese Clinical Trial Registry: ChiCTR2500111204

## 1. Introduction

General anaesthesia is the most widely used anaesthesia method. Over the last 2 decades, scientists have made great progress in understanding the molecular mechanisms of general anaesthesia [1, 2]. However, how general anaesthetics induce reversible loss of consciousness has not been well understood. General anaesthetics have complex effects on permeability transition pore, mitochondrial membrane potential, activity of complex I, complex II and complex III of the respiratory chain and the electron flow along the mitochondrial electron transport chain, which may lead to metabolic inhibition and changes in metabolites and metabolic pathways [3, 4]. Therefore, understanding the association of general anaesthesia and brain metabolism is critical to reveal information about anaesthetic pharmacology. Moreover, metabolic changes might be associated with perioperative brain dysfunction including postoperative delirium (POD), postoperative cognitive dysfunction (POCD) and prolonged anaesthetic recovery, which can cause a prolongation of hospitalisation and worse outcome [5].

Metabolomics, a high‐throughput quantitative approach to study the metabolome, may provide profiling of metabolites in integrated living system responding to factors and give us a new insight into anaesthetic pharmacology and pathological mechanism of perioperative brain dysfunction. Metabolomics analysis has been employed to investigate association between general anaesthesia and alterations in small molecule compounds. Unlike basic research, in which brain of animals can be obtained to detect the alterations of metabolites content after exposure to anaesthetics [6–8], most clinical studies focus on metabolites in serum of patients under anaesthesia [8–11]. Those findings can be affected by other organs and may not specifically reflect cerebral metabolism. Till now, rare attention was paid to the perioperative alterations of metabolites in brain of clinical patients receiving operations under general anaesthesia, which might be attributed to the difficulty in direct measurement of brain metabolism in clinic. However, an alternative is provided to identify uptake or release of metabolites by brain through comparing the abundance of these metabolites in artery versus jugular bulb [12, 13].

In this exploratory study, 20 participants receiving lung surgery under general anaesthesia with sevoflurane or propofol were enrolled. Arterial and jugular venous blood samples were collected perioperatively. Our aim was to establish perioperative changes in cerebral metabolism by arteriovenous gradients of circulating metabolites across brain using liquid chromatography–tandem mass spectrometry (LC‐MS) analysis.

## 2. Methods

### 2.1. Study Design and Participants

This prospective study was conducted at Sichuan Provincial People’s Hospital. Approval for this study (approval No. 2024–175, date of approval March 26, 2024) was granted by the Ethics Committee of Sichuan Provincial People’s Hospital. This study was registered with the Chinese Clinical Trial Registry (registration No. ChiCTR2500111204, principal investigator Peng Li). This study was performed in line with the principles of the Helsinki Declaration. Privacy rights of human subjects have been observed. Written informed consent was obtained from all participants.

Between April 2024 and May 2024, all consecutive individuals scheduled for elective thoracoscopic surgery for unilateral pulmonary lesion were screened. Patients who met the inclusion criteria were contacted by investigators before the surgery to obtain written informed consent. The inclusion criteria were 18 ≤ age ≤ 70 years, 18 kg/m^2^ ≤ body mass index (BMI) ≤ 30 kg/m^2^, general anaesthesia with double‐lumen tube tracheal intubation, single lung lesion with the longest diameter ≤ 2 cm and agreement of patients on participation. The exclusion criteria were pulmonary infection, atelectasis or pneumothorax revealed by preoperative CT, history of lung surgery, forced expiratory volume in 1 s < 60% of predicted value, oxygenation index < 300 mmHg or SpO_2_ < 90% when FiO_2_ was 21%, history of acute upper respiratory tract infection, acute lung injury, acute respiratory distress syndrome or respiratory failure within 3 months prior to surgery, heart failure (The New York Heart Association grade III or IV), history of cerebrovascular accident, history of chronic obstructive pulmonary disease, bronchiectasis, pulmonary bulla or interstitial lung disease, liver failure (Child‐Pugh class B or C), chronic renal failure (glomerular filtration rate < 30 mL/min), non‐cooperation due to mental disease, participation in other clinical trials as subjects within the past 3 months or refusal to participation. Prespecified dropout criteria were duration of one‐lung ventilation (OLV) less than 1 h or more than 6 h, unexpected thoracotomy, withdrawal of consent or serious adverse events. Twenty volunteers were randomised to receive general anaesthesia with sevoflurane (group S, *n* = 10) or propofol (group P, *n* = 10). Figure 1 shows the consort flow diagram for the participants.

**FIGURE 1:**
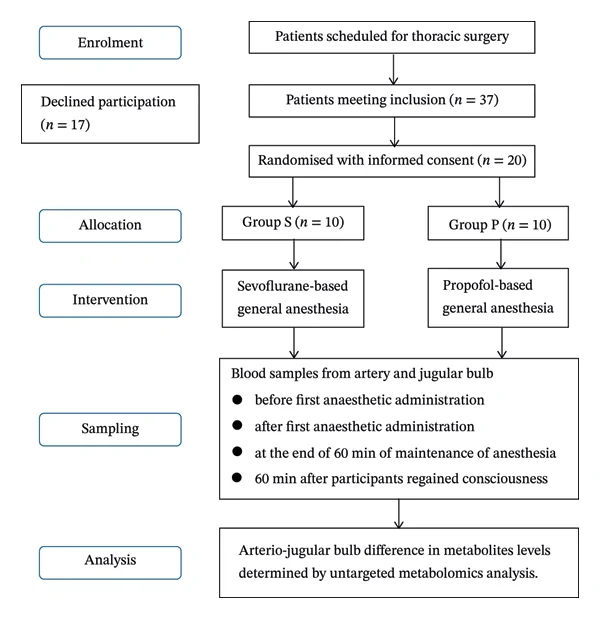
Consort flow diagram for participants. Consort flow diagram shows each stage of the study.

### 2.2. Anaesthetic Protocol and Monitoring

All subjects fasted from the previous midnight. Following peripheral venous catheterisation, catheters were placed in the radial artery and the right jugular bulb, respectively, with the guidance of ultrasound images under local anaesthesia in operation room. Anaesthesia monitoring included electrocardiogram, blood pressure, SpO_2_, end‐tidal carbon dioxide (ETCO_2_) and cerebral state index (CSI). General anaesthesia was induced with sevoflurane (5%–8%, group S) or propofol (1–2.5 mg/kg, group P), followed by administration of sufentanil (0.3–0.5 *μ*g/kg) and cisatracurium (1–1.5 mg/kg) when patients lost consciousness and CSI was between 40 and 60. After bronchial intubation with the guidance of fibre‐optic bronchoscopy, mechanical ventilation was adjusted to have the ETCO_2_ between 30 and 40 mmHg. Anaesthesia was maintained with sevoflurane (1–2.5%, group S) or propofol (5–8 mg kg^−1^·h^−1^, group P), remifentanil (0.1–0.2 *μ*g·kg^−1^·min^−1^) and intermittent doses of cisatracurium with CSI in the range of 40–60. Tropisetron (5 mg) and sufentanil (5–10 *μ*g) were administered at least 1 h after maintenance of general anaesthesia within half an hour before the end of the surgery. Other sedative, general anaesthetic and analgesic were not used during the study. The correct position of the jugular bulb catheter tip was confirmed by postoperative X‐ray. Catheters in radial artery and right jugular bulb were removed 1 h after participants regained consciousness.

### 2.3. Blood Sampling

Blood samples from radial artery and right jugular bulb were collected before first anaesthetic administration (timepoint 0, T0), after first administration of anaesthetic but analgesic or muscle relaxant with patients losing consciousness and CSI in the range of 40–60 (T1), at the end of 60 min of maintenance of general anaesthesia (T2) and 60 min after participants regained consciousness (T3). Blood was collected in EDTA tubes and kept anaerobic and cool. After centrifugation (3500 rpm; 15 min), samples were stored at −80°C until the time of analysis.

### 2.4. LC‐MS‐Based Untargeted Metabolomics Analysis

Briefly, after samples were thawed in an ice water mixture, we mixed 100 *μ*L of serum and 300 *μ*L of a mixture of methanol and acetonitrile (2:1) with rotation for 1 min. Following ultrasound exposure for 1 min in ice water bath, samples were placed at −40°С for 2 h. Subsequently, samples were centrifuged at 13,000 rpm for 10 min at 4°С. Then, the filtered supernatant was transferred to a sampling vial. Equal volumes per sample of methanol‐extracted metabolites were run on a mass spectrometer (Waters ACQUITY UPLC I‐Class plus/Thermo QE HF). Each experimental run included pooled quality control (QC) plasma samples. The sample extract was determined using reversed‐phase chromatography with a mobile phase of water (containing 0.1% formic acid) and acetonitrile at a constant flow rate of 0.35 mL/min. The injection volume was 3 *μ*L, and the temperature of the autosampler was 45°С. The heating electrospray ionisation source parameters were as follows: 35 Arb sheath gas; 8 Arb auxiliary gas; and the spray voltage was 3.8 kV (+)/3.0 kV (−). The capillary temperature was 320°C, while auxiliary gas heater temperature was 325°C.

Multivariate statistical analysis was performed to identify metabolites with significant difference. Potential outliers were identified using principal component analysis (PCA). Metabolites were classified into four levels according to retention time and fragmentation score. Then, partial least squares‐discriminant analysis (PLS‐DA) and orthogonal partial least squares‐discriminant analysis (OPLS‐DA), two supervised multivariate separation methods, were carried out. The model was validated through sevenfold cross‐validation and response permutation testing (*n* = 200) using the fit metrics R2X, R2Y and Q2. Uptake or release of metabolites by brain was determined by the difference in plasma levels between artery and jugular bulb. Systems biology analyses of metabolites were performed using KEGG database. Hypergeometric testing was used to identify pathways where metabolites with significantly different levels were overrepresented.

### 2.5. Statistics

Categorical variables and continuous data, presented as numbers with percentages and mean with standard deviation (SD), respectively, were used to describe baseline characteristics. Categorical variables were compared by Fisher’s exact test, while Student′s *t*‐test was applied to compare continuous data distributed normally, which was confirmed by Kolmogorov–Smirnov test. In addition, Student′s *t*‐test was performed to measure the significance of each metabolite. A *p* < 0.05 was considered statistically significant.

## 3. Results

### 3.1. Baseline Characteristics

A total of 20 volunteers, none of whom withdrew, were enrolled (10 in group S and 10 in group P). The baseline characteristics of the patients are summarised in Table 1. There were no significant differences in sex, age, BMI, American Society of Anesthesiologists (ASA) grade, side of lesion, longest lesion diameter and pathological diagnosis between the two groups.

**TABLE 1:** Baseline characteristics of participants.

Variables	*n* (%) or mean (SD)		*p* value
	Group S	Group P	
Sex			> 0.999
Male	5 (50%)	4 (40%)	
Female	5 (50%)	6 (60%)	
Age (year)	54.10 (6.71)	51.00 (10.46)	0.44
BMI	24.81 (2.97)	23.96 (2.97)	0.53
ASA grade			> 0.999
I or II	8 (80%)	9 (90%)	
III or higher	2 (20%)	1 (10%)	
Side of lesion			0.65
Left	7 (70%)	5 (50%)	
Right	3 (30%)	5 (50%)	
Lesion longest diameter (cm)	1.23 (0.25)	1.22 (0.27)	0.932
Pathology			0.582
Benign	3 (30%)	1 (10%)	
Malignant	7 (70%)	9 (90%)	

Abbreviations: ASA, American Society of Anesthesiologists; BMI, body mass index; SD, standard deviation.

### 3.2. Profiles of Detected Metabolites and Pathways

A total of 3900 metabolites were identified, which consisted of amino acids, peptides and analogues (9.28%), fatty acids and conjugates (9.05%), fatty acid esters (3.08%), carbohydrates and carbohydrate conjugates (2.74%), glycerophosphocholines (2.38%), eicosanoids (2.33%), carbonyl compounds (2.15%), bile acids, alcohols and derivatives (2.1%), benzoic acids and derivatives (1.9%) and others (64.97%). According to retention time and fragmentation score, of these detected metabolites, 537, 814, 373 and 2176 metabolites were at levels 1, 2, 3 and 4, respectively. Only metabolites at levels 1 and 2 were shown subsequently due to the accuracy. In group S, 47, 11, 57 and 45 analytes were identified to be taken up by brain, while 58, 38, 50 and 32 analytes were found to be released from brain at T0, T1, T2 and T3, respectively. In group P, brain obtained 20, 19, 29 and 19 analytes, while it secreted 37, 68, 120 and 87 metabolites at T0, T1, T2 and T3, respectively (Figure 2 and Supporting Tables 1–10).

**FIGURE 2:**
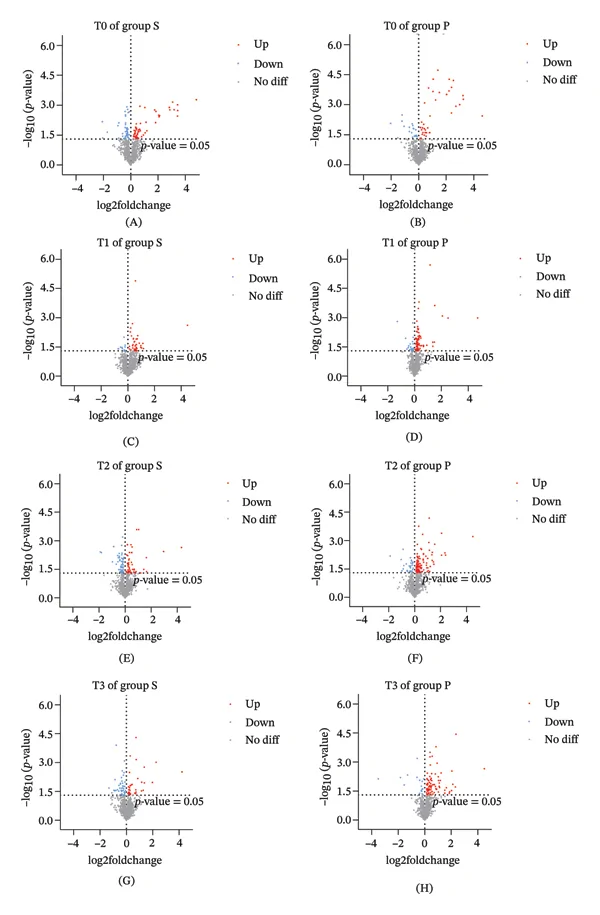
Volcano plot of metabolite abundance in jugular bulb relative to radial artery. Blue and red dots indicate metabolite uptake and release, respectively, in each group at T0 (A and B), T1 (C and D), T2 (E and F) and T3 (G and H).

In group S, concentrations of only 2 and 3 phosphatidylcholines (PCs) significantly altered across cerebral circulation at T0 and T1, respectively; while at T2, up to 15 PCs were taken up but no PC was released by brain; at T3, apart from PC(16:1/0:0), abundance of PCs did not change across cerebral circulation. In group P, abundance of only 2 and 1 PC(s) changed at T0 and T1, respectively; while at T2, in contrast to group S, brain secreted up to 14 PCs but did not take up any PC; and the secretion seemed continuous at T3 (Supporting Table 9). Similarly, alterations of phosphatidylethanolamines (PEs) in group S were opposite to those in group P. At T2 and T3, brain took up PEs in group S, while it secreted these metabolites in group P (Supporting Table 10). Choline was liberated from brain at T1 and T2 in group P, while no liberation was identified at any time point in group S (Supporting Table 10). Isocitrate was released from brain at T2 in group P. In contrast, significant transport of this compound between brain and blood circulation was not detected at any time point in group S (Supporting Table 10). Despite difference in lactic acid at baseline, transport of this metabolite between brain and blood circulation was similar in both groups after anaesthesia induction, with uptake by brain at T2 but no significant transport at T1 and T3 (Supporting Table 10). Changes in other metabolites are shown in Supporting Tables 1–10.

Pathways enriched with significant metabolites are shown in Figures 3 and 4. Key pathways for energy metabolism changed during general anaesthesia. In contrast with T0 and T3, alterations of citrate cycle and pyruvate metabolism were identified at T1 in group S and T2 in group P, while change of glycolysis/gluconeogenesis was identified at T2 in group S (Figures 3 and 4).

FIGURE 3Pathway analysis of significant metabolites in group S. (A, C, E and G) show pathways enriched with metabolites with significantly lower concentration in jugular bulb compared with radial artery at T0, T1, T2 and T3, respectively; while (B, D, F and H) show pathways where metabolites with higher concentration in jugular bulb compared with radial artery at T0, T1, T2 and T3, respectively. Number of significant metabolites enriched in the pathway has been noted at each term. If more than 20 pathways are significantly enriched, only the top 20 are listed.

**Figure:**
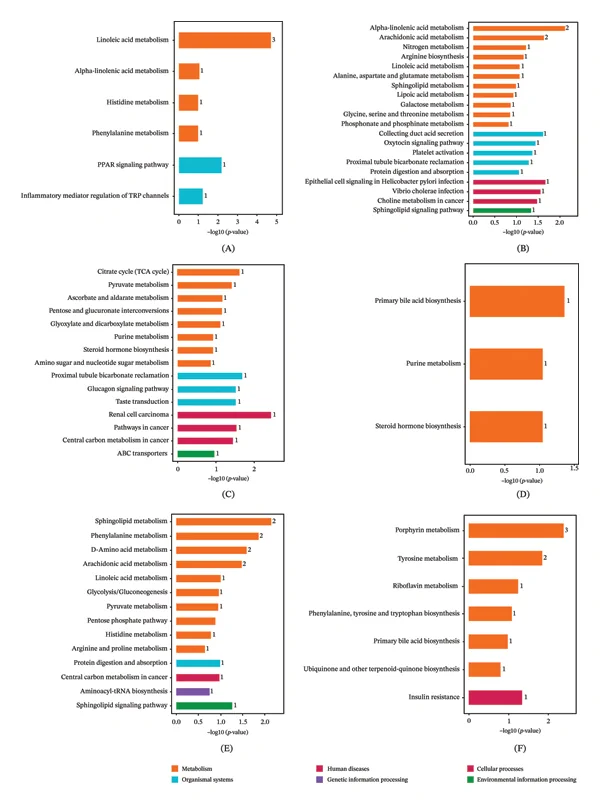

**Figure:**
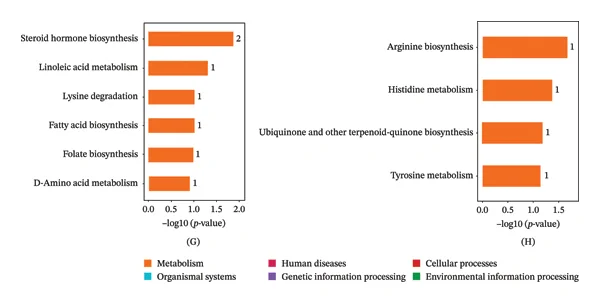

FIGURE 4Pathway enrichment analysis in group P. (A, C, E and G) show pathways enriched with metabolites with significantly lower concentration in jugular bulb compared with radial artery at T0, T1, T2 and T3, respectively; while (B, D, F and H) show pathways where metabolites with higher concentration in jugular bulb compared with radial artery at T0, T1, T2 and T3, respectively. Number of significant metabolites enriched in the pathway has been noted. If more than 20 pathways are statistically significant, only the top 20 are listed.

**Figure:**
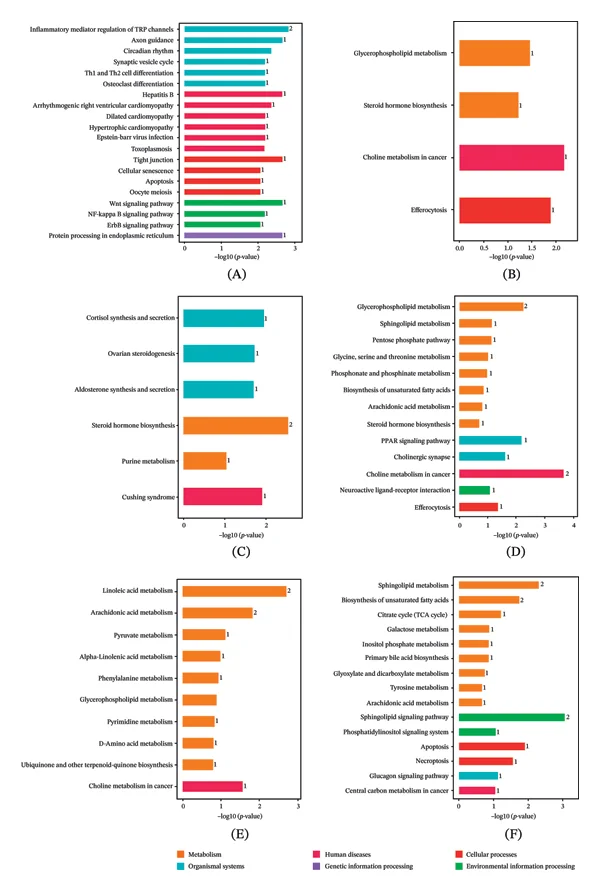

**Figure:**
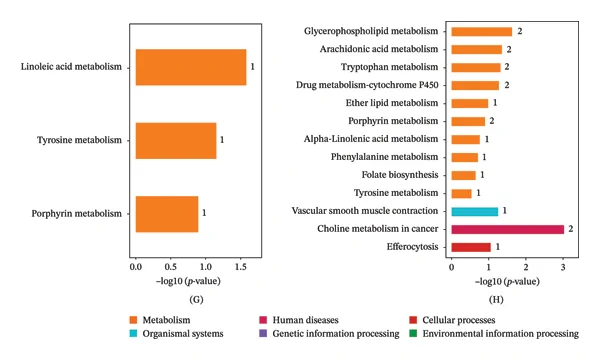

## 4. Discussion

Using peripheral plasma, metabolomics analysis has revealed that exposure to routinely used anaesthetics led to significant and characteristic alterations in the metabolic profile [14, 15]. Moreover, perioperative metabolic abnormalities, including perturbations in tricarboxylic acid (TCA), energy metabolism and amino acid synthesis pathways in addition to *ω*3 and *ω*6 fatty acids deficiency, may play a role in POD [9–11]. However, metabolic changes in brain tissue and peripheral blood are not completely consistent [8]. Therefore, cerebrospinal fluid is used in metabolomic research. One of these studies has reported that altered glucose utilisation, and a shift to ketone body metabolism may occur in patients who underwent surgery for hip fracture under spinal anaesthesia and developed delirium [16]. Moreover, the pentose phosphate pathway may be a crucial target for sedatives which alter brain metabolism [17]. However, transport of metabolites between brain and blood circulation during general anaesthesia remains poorly understood. In this study, we evaluated the perioperative cerebral metabolic profiles in participants receiving thoracic surgery under anaesthesia with sevoflurane or propofol by comparing the abundance of metabolites in the artery versus jugular bulb.

To our knowledge, there was no previous clinical study detecting perioperative metabolic changes of PC or PE. Interestingly, our findings revealed that anaesthesia with sevoflurane might lead to uptake of both PC and PE by brain while anaesthesia with propofol might result in secretion of those two compounds from brain. These two most abundant membrane phospholipids serve a neuroprotective role by providing an important component of cellular membranes and acting as reservoirs for second messengers [18]. PC can be synthesised from PE [19]. Accelerated breakdown of PC and PE is thought to be a predominant brain metabolic defect in Alzheimer’s disease (AD), while administration of PC, serving as an essential portion of the total choline pool, can improve memory in mice with dementia [18, 20, 21]. Additionally, PCs and PEs are precursors of phosphatidylserine, which is a major anionic phospholipid class in brain, and endocannabinoids, which are endogenous lipid mediators playing a critical role in normal neuronal excitation and inhibition through acting on presynaptically expressed CB1 receptors in brain [22–25]. Endocannabinoids play an essential role in the interaction between prefrontal cortex, amygdala and hippocampus [22]. Taken together, sevoflurane and propofol led to different trends in metabolic changes of PC and PE, which are two basic components of neurons and have a critical impact on brain function.

Choline is an essential nutrient that the organs especially brain need in vast quantities for cell membrane synthesis, epigenetic modification and neurotransmission. Choline can be both product and precursor of PC. Nevertheless, effects of general anaesthetics on metabolism of choline in brain remain controversial. Söbbeler et al. detect a reduction in total choline in brain of Beagle dogs receiving volatile anaesthetics by proton magnetic resonance spectroscopy [26]. However, Boretius et al. observe that isoflurane induces an increase of choline‐containing compounds by 20% in brain of mice [27]. Ngai et al. demonstrate that ketamine increases the concentration of choline in hippocampus of rat while halothane and enflurane do not affect it [28]. Griffiths et al. report that volatile anaesthetic agents inhibit choline uptake into rat synaptosomes [29]. Yang et al. find enhanced expression of both choline acetyltransferase and acetylcholinesterase in ageing model rats receiving sevoflurane [30]. Interestingly, Hu et al. report that sevoflurane increases the expression of choline acetyltransferase but decreases the expression of acetylcholinesterase in hippocampal CA1 region of rat with shock [31]. In this study, we failed to find that brain statistically significantly obtained or released choline in patients receiving anaesthesia with sevoflurane. It has been reported in the literature that propofol reduces the levels of choline in the brain of healthy volunteers [32]. An in vitro study demonstrates that propofol reduces the activity of acetylcholinesterase and butyrylcholinesterase by epigenetic modifications [33]. In our study, we found that brain releases choline in patients receiving propofol during anaesthesia induction and maintenance. Taken together, we hypothesise that reduction of choline concentration in brain following administration of propofol may be partially induced by both inhibited generation and increased transport to blood circulation.

Choline acetyltransferase catalyses the addition of an acetyl group to choline to generate acetylcholine. After acetylcholine is released into the synaptic cleft for neurotransmission, it is ultimately hydrolysed to acetate and choline by acetylcholinesterase, thereby enabling choline re‐uptake into the presynaptic neurons to be recycled into acetylcholine [34]. Cholinergic neurons in the nucleus basalis magnocellularis are important in the behavioural responses to general anaesthetics [35]. Moreover, central cholinergic neuronal degeneration and abnormality of acetylcholine metabolism are associated with the development of POCD and delayed neurocognitive recovery [36]. Thus, further research may be required to evaluate whether changes in choline metabolism play a role in anaesthetic pharmacology and pathogenesis of perioperative brain dysfunction.

Isocitrate, known as one of the intermediates of TCA cycle, is associated with mitochondrial function and brain disorder. The activity of isocitrate dehydrogenase decreases by 27% in AD [37]. Interestingly, patients with increased serum isocitrate concentrations have greater incidence of dementia, but the cognitive function of AD patients with higher serum isocitrate concentrations decreases less rapidly [38, 39]. Compared with isoflurane, propofol decreases the levels of intermediates within the first span of the TCA cycle that extends from citrate to *α*‐ketoglutarate and includes isocitrate in parietal cortex of immature swine [40]. In this study, we found that propofol might enable isocitrate in brain to enter blood circulation, while sevoflurane did not have this effect.

The accumulation of lactate in ischaemic tissues has established lactate’s reputation as a deleterious waste product. Postoperative elevated lactate levels in blood and parietal cortex are associated with an increased risk of POD [41–43]. Similarly, there is a positive correlation between lactate concentration in cerebrospinal fluid and POD severity [44]. Moreover, it has been reported that arterio‐jugular venous lactate difference is one of the predictors of POCD after off‐pump coronary artery bypass grafting [45]. Influence of lactate on postoperative cognitive function is complicated. On the one hand, lactate improves postoperative cognitive function by attenuating oxidative stress and neuroinflammation, and promoting hippocampal neurogenesis and synaptic plasticity [44, 46, 47]. On the other hand, lactate in neurons may lead to synaptic impairment, insufficient PTEN‐induced kinase 1‐dependent mitophagy and hyperactive caspase‐3/gasdermin E‐dependent pyroptosis [48]. In addition, blood lactate can enter the human brain as an important energy source [49]. Perioperative alterations of metabolism of lactate remain controversial. Dumaresq et al. demonstrate that plasma lactate concentrations remain unchanged in children receiving elective cardiac surgery with cardiopulmonary bypass under anaesthesia with either sevoflurane or propofol, while Indra et al. find that serum lactate value decreases in paediatric patients undergoing outpatient procedural sedation with propofol [50, 51]. Jacob et al. reveal that compared to propofol, sevoflurane induces greater lactate accumulation in parietal cortex [43]. Furthermore, Singh et al. report that brain releases lactate in patients receiving off‐pump coronary artery bypass grafting [45]. Inconsistently, our results showed that lactate entered brain intraoperatively in patients receiving lung surgery under general anaesthesia with sevoflurane or propofol. Taken together, lactate is a biomarker of POD and POCD, and perioperative changes in lactate metabolism and its impact on perioperative neurocognitive disorder may both be very complicated.

Our pathway enrichment analysis revealed that key pathways for energy metabolism including citrate cycle, pyruvate metabolism and glycolysis/gluconeogenesis were altered during general anaesthesia. Postoperative abnormal citrate cycle pathway is confirmed in POD, and energy metabolism dysregulation is thought to be capable of triggering this disease [9]. Postoperative dysregulation of citrate cycle was not detected in this study because few of our patients were diagnosed with POD. Further research may be required to explore the complex interactions between metabolic pathways and other physiological signal transduction networks using strategies including integrative analysis of multi‐omics [52].

A few limitations must be addressed. First, this is a single‐centre study with relatively small sample size and admission bias may be present. Second, absolute concentrations of these metabolites were not determined, which might lead to failure of identification of some other significant metabolic changes. Additionally, it was difficult to differentiate the effects of propofol from those of the accompanying lipid emulsion on metabolism.

## 5. Conclusion

In summary, we measured the uptake and secretion of circulating metabolites by brain in patients receiving lung surgery under general anaesthesia with sevoflurane or propofol. Several potential critical alterations in metabolites including PC, PE, choline, isocitrate and lactate and pathways were identified. The present data may provide a framework of perioperative cerebral metabolism and a new direction for further studies in pharmacology of general anaesthetics and pathogenesis of perioperative brain dysfunction.

## Author Contributions

Conceptualisation: Peng Li.

Methodology: Chenghong Wang.

Software: Sike Ma.

Validation: Xudan Xing.

Formal analysis: Sike Ma.

Investigation: Qian Lei.

Data curation: Yi Yang.

Resources: Qian Lei.

Writing–original draft: Chenghong Wang.

Writing–review and editing: Qian Liu.

Visualisation: Qian Liu.

Supervision: Juan Liao.

Project administration: Juan Liao.

Funding acquisition: Peng Li.

## Funding

This work was supported by the National Natural Science Foundation of China (grant number 82170634 PL), Sichuan Provincial Natural Science Foundation (grant number 2026NSFSC0541 PL) and the Major Science and Technology Project of the Sichuan Provincial Department of Science and Technology (grant number 2025ZDZX0129).

## Conflicts of Interest

The authors declare no conflicts of interest.

## Supporting Information

Additional supporting information can be found online in the Supporting Information section.

## Supporting information


**Supporting Information** Supporting Table 1. Fold changes of levels of amino acids, peptides and analogues with significant difference between artery and jugular bulb. Supporting Table 2. Fold changes of levels of benzoic acids and derivatives with significant difference between artery and jugular bulb. Supporting Table 3. Fold changes of levels of bile acids, alcohols and derivatives with significant difference between artery and jugular bulb. Supporting Table 4. Fold changes of levels of carbohydrates and carbohydrate conjugates with significant difference between artery and jugular bulb. Supporting Table 5. Fold changes of levels of carbonyl compounds with significant difference between artery and jugular bulb. Supporting Table 6. Fold changes of levels of eicosanoids with significant difference between artery and jugular bulb. Supporting Table 7. Fold changes of levels of fatty acid esters with significant difference between artery and jugular bulb. Supporting Table 8. Fold changes of levels of fatty acids and conjugates with significant difference between artery and jugular bulb. Supporting Table 9. Fold changes of levels of glycerophosphocholines with significant difference between artery and jugular bulb. Supporting Table 10. Fold changes of levels of compounds in other subclasses with significant difference between artery and jugular bulb.

## Data Availability

The data that support the findings of this study are available on request from the corresponding author. The data are not publicly available due to privacy or ethical restrictions.
