# Supplementary material for: Perioperative Cerebral Metabolism in Patients Under General Anaesthesia With Sevoflurane or Propofol: Evaluation of the Arterial and Jugular Bulb Metabolome
Source: Anesthesiol Res Pract. 2026 Oct 23;2026:8559251. doi: 10.1155/anrp/8559251 (PMC13693408; doi:10.1155/anrp/8559251)
Supplement: Supplementary file 1 [file ANRP-2026-8559251-s001.docx]

Supplementary Table 1. Fold changes of levels of amino acids, peptides, and analogues with significant difference between artery and jugular bulb.

| Metabolites | Fold change of metabolite levels | | | | | | | |
| --- | --- | --- | --- | --- | --- | --- | --- | --- |
|  | Group S | | | | Group P | | | |
|  | T0 | T1 | T2 | T3 | T0 | T1 | T2 | T3 |
| (1R,2S)-2-aminocyclopentanecarboxylic acid |  |  |  | 0.92 |  |  |  | 1.23 |
| (2E)-decenoyl-ACP |  |  |  | 0.79 |  |  |  |  |
| (S)-2-acetamido-3-(4-chlorophenyl)propanoic acid |  |  |  |  |  |  |  | 1.12 |
| 2-phenylglycine | 3.50 |  |  |  |  |  |  |  |
| 4-hydroxy-2-nonenal-[Cys-Gly] conjugate |  |  | 2.04 |  |  |  | 2.55 | 1.86 |
| β-leucine |  |  |  |  |  | 0.80 |  |  |
| γ-aminobutyric acid-β-xanthin |  |  |  |  |  |  |  | 1.74 |
| γ-glutamylglutamine |  |  |  | 1.35 |  |  |  |  |
| γ-glutamylleucine |  | 0.87 |  |  |  |  |  |  |
| γ-glutamyltyrosine |  |  |  |  |  | 1.58 |  |  |
| acetyl-DL-leucine |  |  |  |  |  |  | 0.82 | 1.42 |
| acetyl-L-lysine |  |  | 1.49 | 1.68 |  |  |  |  |
| Brivaracetam |  |  |  |  |  |  | 0.91 |  |
| caffeoyl tyrosine |  |  |  | 0.87 |  |  |  | 1.47 |
| cyclo(Pro-Leu) |  |  |  | 0.86 |  |  | 1.19 |  |
| D-pipecolinic acid |  | 0.82 |  |  |  |  |  |  |
| D-ornithine |  |  |  |  |  | 0.92 |  |  |
| glutamylvaline |  |  |  |  |  |  | 0.83 |  |
| homocysteine thiolactone | 1.29 |  |  |  |  | 0.88 |  |  |
| hydroxytyrosyl acetate |  |  |  |  |  |  |  | 1.46 |
| isoleucyl-tyrosine |  |  |  |  |  | 1.19 |  |  |
| leucylphenylalanine |  |  | 1.45 |  |  |  |  |  |
| L-proline |  |  | 0.83 |  |  |  |  |  |
| N-(3-hydroxy-eicosanoyl)-homoserine lactone |  |  |  |  |  | 1.27 |  |  |
| N-acetyl-DL-serine | 0.80 |  |  |  |  |  |  |  |
| N-acetyl-L-phenylalanine |  |  | 0.79 |  |  |  | 0.80 | 1.35 |
| N-acetyl-L-tyrosine |  |  |  | 1.21 |  |  |  | 1.35 |
| N-acetyl-L-tryptophan |  |  |  |  |  |  |  | 1.23 |
| N-acetyl-ornithine |  |  |  | 1.21 |  |  |  |  |
| N-acetyl-S-methylcysteine |  |  |  |  | 1.16 |  |  |  |
| N-lauroylsarcosine | 0.70 |  |  |  |  |  |  |  |
| N-linoleoyl leucine | 0.77 |  |  |  |  |  |  |  |
| N-methylalanine |  |  | 0.81 |  |  |  |  |  |
| N-palmitoyl alanine |  |  | 1.20 |  |  |  |  |  |
| N-palmitoyl glutamine |  |  |  |  |  | 1.87 |  |  |
| N-palmitoyl proline |  |  |  | 0.55 |  |  |  |  |
| N-oleoyl L-serine |  |  |  | 1.14 |  |  |  |  |
| phenylalanylmethionine |  | 0.61 |  |  |  |  |  |  |
| Pregabalin |  |  |  | 2.21 |  |  |  |  |
| prolylphenylalanine |  |  |  |  |  |  | 1.21 |  |
| thyroxine | 0.85 | 1.17 |  |  |  |  |  |  |
| Trp-Gly-Glu |  |  |  |  |  | 1.19 |  |  |
| Tyr-Tyr |  |  |  |  |  |  | 0.68 |  |
| Z-Leu-Leu-Norvalinal |  | 1.42 |  | 1.23 |  | 1.25 |  |  |

Fold change <1 and >1 indicates uptake and secretion of circulating metabolites by brain, respectively.

Supplementary Table 2. Fold changes of levels of benzoic acids and derivatives with significant difference between artery and jugular bulb.

| Metabolites | Fold change of metabolite levels | | | | | | | |
| --- | --- | --- | --- | --- | --- | --- | --- | --- |
|  | Group S | | | | Group P | | | |
|  | T0 | T1 | T2 | T3 | T0 | T1 | T2 | T3 |
| (Z)-2,4-dihydroxy-6-(8-pentadecenyl)benzoic acid |  |  |  |  | 0.43 |  |  |  |
| 2-chlorobenzoic acid |  |  |  |  |  |  |  | 0.88 |
| 2-methylbenzoic acid |  |  |  | 1.18 |  |  |  | 0.90 |
| 3-(2-ethyl-5-oxohexyl)phthalic acid | 0.55 |  |  |  |  |  |  |  |
| 3-hexenyl salicylic acid |  |  |  |  | 0.81 |  |  |  |
| 3-polyprenyl-4-hydroxy-5-methoxybenzoate | 1.38 |  |  |  |  |  |  |  |
| 3,5-diisopropylsalicylic acid |  | 1.39 |  |  |  |  |  |  |
| Aspirin |  |  | 0.55 | 0.67 | 0.63 |  | 0.54 |  |
| isoamyl p-anisate |  |  |  |  |  | 1.12 | 1.31 | 1.14 |
| isoamyl salicylate |  |  |  |  |  | 1.05 |  |  |
| Janohigenin 21-0 |  |  | 0.92 |  |  |  |  |  |
| Janohigenin 23-0 | 1.21 |  |  |  |  |  |  |  |
| Janohigenin 25-0 | 1.25 |  |  |  |  |  |  |  |
| monooctyl phthalate |  |  |  |  | 0.43 |  |  |  |
| N'-benzoyl-N,N'-dimethylbenzohydrazide |  |  |  |  | 1.78 |  |  |  |
| Naepaine |  |  |  |  | 0.70 |  |  |  |

Fold change <1 and >1 indicates uptake and secretion of circulating metabolites by brain, respectively.

Supplementary Table 3. Fold changes of levels of bile acids, alcohols and derivatives with significant difference between artery and jugular bulb.

| Metabolites | Fold change of metabolite levels | | | | | | | |
| --- | --- | --- | --- | --- | --- | --- | --- | --- |
|  | Group S | | | | Group P | | | |
|  | T0 | T1 | T2 | T3 | T0 | T1 | T2 | T3 |
| 3-dehydroteasterone |  |  |  |  |  |  |  | 1.75 |
| 3a,7a-dihydroxy-5b-cholestan-26-al |  |  |  |  |  |  | 1.12 |  |
| 5β-Cholane-3α,24-diol |  |  | 0.88 |  |  |  |  |  |
| 6β,12α-dihydroxy-3-oxo-5β-cholan-24-oic acid |  | 2.05 |  |  |  |  |  |  |
| 7α-hydroxy-3-oxocholest-4-en-27-oic acid |  |  | 1.21 |  |  | 1.11 | 1.16 |  |
| ω-muricholic acid | 0.66 |  |  |  |  |  | 1.62 |  |
| chenodeoxycholic acid sulfate |  |  | 2.29 |  |  |  |  |  |
| deoxycholic acid glycine conjugate |  |  |  |  |  |  | 1.34 |  |
| deoxycholylphenylalanine |  |  |  | 0.80 |  |  | 1.19 |  |
| hyodeoxycholic acid |  |  | 1.27 |  |  |  |  |  |

Fold change <1 and >1 indicates uptake and secretion of circulating metabolites by brain, respectively.

Supplementary Table 4. Fold changes of levels of carbohydrates and carbohydrate conjugates with significant difference between artery and jugular bulb.

| Metabolites | Fold change of metabolite levels | | | | | | | |
| --- | --- | --- | --- | --- | --- | --- | --- | --- |
|  | Group S | | | | Group P | | | |
|  | T0 | T1 | T2 | T3 | T0 | T1 | T2 | T3 |
| (E)-2-O-cinnamoyl-β-D-glucopyranose | 1.17 |  |  |  |  |  |  |  |
| 2S-hydroxy-3-(10Z-tetradecenoyloxy)-propanoic acid |  |  |  |  |  |  | 1.24 |  |
| 4-methoxybenzyl glucoside |  |  |  |  | 0.75 |  |  |  |
| 5-deoxy-D-ribose |  |  |  |  |  |  |  | 0.88 |
| β-D-galactosamine |  |  |  |  |  |  | 0.51 |  |
| γ-CEHC glucuronide |  |  |  |  |  |  |  | 1.13 |
| Cichorioside F |  |  | 1.70 |  |  |  |  |  |
| D-glucose |  |  | 0.75 |  |  |  |  |  |
| D-ribonolactone |  |  | 1.14 |  |  |  |  |  |
| L-(+)-Arabinose |  | 0.74 |  |  |  |  |  |  |
| Melilotoside |  |  |  | 0.91 |  |  |  |  |
| Propofol glucuronide | 0.24 |  |  |  |  | 4.19 |  |  |
| Torachrysone 8-β-gentiobioside |  |  |  |  |  |  | 1.21 |  |

Fold change <1 and >1 indicates uptake and secretion of circulating metabolites by brain, respectively.

Supplementary Table 5. Fold changes of levels of carbonyl compounds with significant difference between artery and jugular bulb.

| Metabolites | Fold change of metabolite levels | | | | | | | |
| --- | --- | --- | --- | --- | --- | --- | --- | --- |
|  | Group S | | | | Group P | | | |
|  | T0 | T1 | T2 | T3 | T0 | T1 | T2 | T3 |
| 1-(2,6-dihydroxy-4-methoxyphenyl)-6Z,9Z,12Z,15Z-octadecatetraen-1-one | 1.29 |  |  |  | 1.34 |  |  |  |
| 2-(5,8-tetradecadienyl)cyclobutanone | 3.35 |  |  |  | 2.34 | 0.00 |  | 1.64 |
| 2-methyl-3-oxo-propanoic acid |  |  |  |  |  |  | 0.85 |  |
| 2,6,6-trimethyl-2-cyclohexene-1,4-dione |  |  |  |  | 0.89 |  |  |  |
| 3-(methylthio)propanal |  |  | 0.88 |  |  |  |  |  |
| 4-nonenal |  |  | 0.92 |  |  |  |  |  |
| 5-ethylpentadecane-2,4-dione |  | 1.14 |  |  |  |  |  | 1.31 |
| 5R-hydroxy-4S-methylheptan-3-one |  |  |  | 0.79 |  |  |  | 1.35 |

Fold change <1 and >1 indicates uptake and secretion of circulating metabolites by brain, respectively.

Supplementary Table 6. Fold changes of levels of eicosanoids with significant difference between artery and jugular bulb.

| Metabolites | Fold change of metabolite levels | | | | | | | |
| --- | --- | --- | --- | --- | --- | --- | --- | --- |
|  | Group S | | | | Group P | | | |
|  | T0 | T1 | T2 | T3 | T0 | T1 | T2 | T3 |
| 5S-HETE |  |  | 0.73 |  | 0.00 |  | 2.30 | 1.57 |
| 12(S)-HETrE |  |  |  |  |  | 1.18 |  |  |
| 12-keto-10,11,14,15-tetrahydro-LTB4 |  |  |  |  |  | 1.35 |  |  |
| 13-epi-12-oxo phytodienoic acid |  | 1.08 |  |  |  |  |  |  |
| 14,15-dihydroxy-5,8,10,12-eicosatetraenoic acid | 0.83 |  |  |  |  |  |  |  |
| 15-dihydroxyeicosatrienoic acid |  |  |  | 0.72 |  |  |  |  |
| 15S-HpEDE |  |  |  |  |  |  |  | 1.14 |
| 18-hydroxy-arachidonic acid |  | 1.54 |  |  |  |  |  |  |
| Auricolic acid |  |  |  |  |  |  | 1.82 |  |
| diglyceride(2:0/PGJ2/0:0) |  |  |  |  |  |  |  | 1.23 |
| dinor-PGE2 |  |  | 0.83 |  |  |  |  |  |
| phosphatidylcholine(PGJ2/17:0) |  |  |  | 1.28 |  |  |  | 0.67 |
| prostaglandin H2 | 1.32 |  |  |  |  |  |  |  |
| thromboxane B3 |  |  |  |  |  | 1.21 |  | 1.30 |

Fold change <1 and >1 indicates uptake and secretion of circulating metabolites by brain, respectively.

Supplementary Table 7. Fold changes of levels of fatty acid esters with significant difference between artery and jugular bulb.

| Metabolites | Fold change of metabolite levels | | | | | | | |
| --- | --- | --- | --- | --- | --- | --- | --- | --- |
|  | Group S | | | | Group P | | | |
|  | T0 | T1 | T2 | T3 | T0 | T1 | T2 | T3 |
| (5,7)-decadienoylcarnitine |  |  |  |  |  |  | 1.28 |  |
| [8]-gingerdiol |  |  |  |  |  |  |  | 0.67 |
| 3-icosa-8,11,14-trienoylcarnitine |  |  |  | 0.90 |  |  |  |  |
| 5-methylheptadecanoylcarnitine |  | 1.28 |  |  |  |  |  |  |
| 7-methyloctanoylcarnitine |  |  |  |  | 1.40 |  |  |  |
| 17-methylnonadecanoylcarnitine |  |  |  |  |  | 1.19 |  |  |
| arachidonic acid methyl ester |  |  |  |  |  |  |  | 1.65 |
| docosahexaenoic acid methyl ester |  |  |  |  |  |  | 1.58 | 2.05 |
| isoachifolidiene |  |  |  |  |  |  | 1.87 |  |
| octadecyl fumarate |  |  | 1.26 |  |  |  |  |  |
| Siegesbeckin G |  |  |  |  |  |  | 1.12 |  |
| Tiglyl carnitine |  |  |  |  |  |  | 2.23 | 2.85 |

Fold change <1 and >1 indicates uptake and secretion of circulating metabolites by brain, respectively.

Supplementary Table 8. Fold changes of levels of fatty acids and conjugates with significant difference between artery and jugular bulb.

| Metabolites | Fold change of metabolite levels | | | | | | | |
| --- | --- | --- | --- | --- | --- | --- | --- | --- |
|  | Group S | | | | Group P | | | |
|  | T0 | T1 | T2 | T3 | T0 | T1 | T2 | T3 |
| (4E,7E,10Z,13E,16E,19E)-docosa-4,7,10,13,16,19-hexaenoic acid |  |  | 1.16 |  |  |  |  |  |
| (9S,10S)-9,10-dihydroxyoctadecanoic acid | 0.76 |  |  |  |  |  |  | 0.84 |
| (13E,15E)-octadeca-13,15-dien-9,11-diynoic acid |  | 1.27 | 1.14 |  |  |  |  |  |
| 2-methoxytetradecanoic acid | 0.88 |  |  |  |  |  |  |  |
| 2,10-dihydroxy-4,6,8-decatriynoic acid |  | 0.87 |  |  |  |  |  |  |
| 3-hydroxy-tetradecanedioic acid |  | 0.82 |  |  |  | 1.24 |  |  |
| 3-hydroxy-tridecanoic acid | 0.75 |  | 0.81 |  |  |  |  |  |
| 3,4-dimethyl-5-carboxyethyl-2-furanpentanoic acid |  |  |  |  |  |  |  | 0.77 |
| 4-[3]-ladderane-butanoic acid |  |  |  |  |  |  | 1.07 |  |
| 4-octadecynoic acid |  |  |  |  | 1.16 |  |  |  |
| 4,5-dehydro docosahexaenoic acid |  |  |  | 0.58 |  |  |  |  |
| 4,5-leukotriene A4 |  | 1.28 |  |  |  |  |  | 1.12 |
| 4,8,12,16-hexadecatetraenoic acid |  |  |  |  |  |  | 1.10 |  |
| 4-hydroxy-4-methyl-5-hexenoic acid gamma lactone |  | 0.83 |  |  |  |  |  |  |
| 5Z,8Z,11Z-eicosatrienedioic acid |  |  |  |  | 0.92 |  |  |  |
| 6Z,9Z,12Z,15Z,18Z-tetracosapentaenoic acid |  |  |  |  | 1.48 |  | 1.44 |  |
| 7(8)-EpDPE |  | 1.16 |  |  |  |  |  |  |
| 7Z,10Z,13Z,16Z,19Z-eicosapentaenoic acid |  |  |  |  |  |  | 1.31 |  |
| 8-hydroxy-10,12-octadecadiynoic acid |  |  |  |  |  | 1.44 |  |  |
| 8-tetradecynoic acid |  | 1.53 |  |  |  |  |  |  |
| 8,11,14,17-eicosatetraenoic acid |  |  |  |  |  | 1.14 |  |  |
| 8E-heptadecenedioic acid |  |  |  |  |  |  | 1.1 |  |
| 8R,9R-epoxy-17-octadecen-4,6-diynoic acid |  |  | 0.82 |  |  |  |  |  |
| 8Z,12E,14Z-eicosatrienoic acid | 1.35 |  |  |  |  |  |  |  |
| 9-hydroperoxy-12,13-epoxy-10-octadecenoic acid |  |  |  |  |  |  | 1.13 |  |
| 9-tetradecynoic acid | 0.87 |  |  |  |  |  |  |  |
| 9,10-DiHOME(12) | 0.82 |  |  |  |  |  |  |  |
| 9,10,13-TriHOME(11) | 0.24 |  | 0.27 | 0.40 | 0.25 |  | 0.27 | 0.28 |
| 9,12-hexadecadienoic acid |  |  | 1.37 |  | 1.72 |  | 1.60 |  |
| 9,13-DiHOME(10) | 0.55 |  |  |  | 1.75 |  |  | 0.84 |
| 9E-octadecen-12-ynoic acid |  |  |  |  |  | 1.17 |  |  |
| 9S,10S,11R-trihydroxy-12Z-octadecenoic acid | 0.30 |  | 0.28 |  | 0.35 |  | 0.49 | 0.34 |
| 10-keto palmitic acid |  |  | 0.88 |  |  |  |  |  |
| 11-(3,4-dimethyl-5-propylfuran-2-yl)-undecanoic acid |  |  |  |  |  |  |  | 1.29 |
| 11-cycloheptyl,2R-hydroxyundecanoic acid |  |  |  |  |  | 1.10 |  |  |
| 11-methoxy-nonadecanoic acid |  |  | 0.76 |  |  |  |  |  |
| 12-hydroxy-8E,10E-heptadecadienoic acid | 1.32 |  |  |  |  |  |  |  |
| 12-hydroxy-9-octadecynoic acid | 0.93 |  |  |  |  |  |  |  |
| 12-tetradecynoic acid |  |  |  |  |  |  |  | 1.44 |
| 13-(3-pentyloxiran-2-yl)trideca-2,4,6-trienoic acid |  |  | 1.16 |  |  |  |  |  |
| 14,15-leukotriene A4 |  |  | 1.43 |  |  |  |  |  |
| 15-hydroxy-hexadecanoic acid |  |  | 0.84 |  |  |  |  |  |
| 19-HOME(9Z) | 0.91 |  |  |  |  |  |  |  |
| 20-HDoHE |  | 1.66 |  |  |  |  |  |  |
| 21-hydroxy-heneicosanoic acid |  |  |  |  |  |  |  | 1.51 |
| adrenic acid |  |  | 1.17 |  |  |  |  | 1.14 |
| Aleprylic acid |  |  |  |  |  |  |  | 0.84 |
| athanacalvic acid | 0.82 |  | 0.84 |  |  |  |  |  |
| Azelaic acid |  |  |  |  |  |  | 0.90 |  |
| capric acid |  |  |  | 0.67 |  |  |  |  |
| Cladosporacid E |  |  |  |  |  | 0.92 |  |  |
| cyclopentanetridecanoic acid |  |  |  | 0.72 |  |  |  |  |
| Dicranin |  |  | 1.24 |  |  |  |  |  |
| docosapentaenoic acid (22n-3) |  |  | 1.13 |  |  |  |  |  |
| eicosapentaenoic acid | 1.28 |  |  |  |  |  | 1.23 | 1.16 |
| hexacosanedioic acid |  |  |  |  |  |  |  | 1.39 |
| isoketocamphoric acid |  |  |  |  |  | 0.82 |  |  |
| lycocasuarinen acid E |  | 1.60 |  |  |  |  |  |  |
| Juniperic acid |  |  |  |  |  | 1.02 |  |  |
| Lesquerolic acid |  |  |  | 0.90 |  |  |  |  |
| Margaric acid |  |  |  |  |  | 1.20 |  |  |
| Medica 16 |  | 1.21 |  |  |  |  |  |  |
| Nonic acid |  |  |  |  |  |  |  | 0.88 |
| octadec-11-en-9-ynoic acid | 1.57 |  |  |  | 1.57 |  |  |  |
| Petroselinic acid | 1.32 |  |  |  |  | 1.14 |  |  |
| Phloionolic acid | 0.83 |  |  |  |  | 1.14 |  | 1.22 |
| Protectin D1n-3 DPA |  |  |  |  |  |  | 1.31 |  |
| tetracosanedioic acid |  |  |  | 0.85 |  |  |  |  |
| tetranor-12(R)-HETE |  | 1.48 |  |  |  |  |  |  |
| tetranorprostanedioic acid |  |  |  |  |  |  | 1.09 |  |
| Wuhanic acid |  |  |  |  |  | 1.15 |  |  |

Fold change <1 and >1 indicates uptake and secretion of circulating metabolites by brain, respectively.

Supplementary Table 9. Fold changes of levels of glycerophosphocholines with significant difference between artery and jugular bulb.

| Metabolites | Fold change of metabolite levels | | | | | | | |
| --- | --- | --- | --- | --- | --- | --- | --- | --- |
|  | Group S | | | | Group P | | | |
|  | T0 | T1 | T2 | T3 | T0 | T1 | T2 | T3 |
| 1-myristoyl-sn-glycero-3-phosphocholine |  |  |  |  |  |  |  | 1.11 |
| 1-O-(2-methoxyhexadecyl)-sn-glycerol-3-phosphocholine |  |  |  |  |  |  | 2.06 |  |
| 2-lysophosphatidylcholine |  |  | 0.87 |  |  |  | 1.13 |  |
| Butanoyl PAF |  |  | 0.80 |  |  |  |  | 1.40 |
| lysoPG(20:3/0:0) |  |  |  |  |  |  |  | 1.25 |
| **PC(0:0/14:0)** |  |  |  |  |  |  | **1.13** | **1.15** |
| **PC(0:0/18:0)** |  |  | **0.94** |  |  |  | **1.11** |  |
| **PC(13:0/0:0)** |  | **2.18** |  |  |  |  |  |  |
| **PC(14:0/2:0)** |  |  | **0.81** |  |  |  | **1.21** |  |
| **PC(16:0/0:0)[rac]** |  |  | **0.84** |  |  |  |  |  |
| **PC(16:0/5:0(CHO))** |  |  |  |  |  |  | **1.12** | **1.14** |
| **PC(16:0/18:1-O(9S,10R))** |  | **1.34** |  |  |  |  |  |  |
| **PC(16:1/0:0)** |  |  |  | **0.96** |  |  |  | **1.13** |
| **PC(17:0/0:0)** |  |  | **0.90** |  |  |  | **1.17** |  |
| **PC(17:0/17:0)** |  |  |  |  | **0.86** |  |  |  |
| **PC(17:1/0:0)** |  |  | **0.87** |  |  |  |  | **1.11** |
| **PC(18:0/14:1)** | **1.45** |  |  |  |  |  |  |  |
| **PC(18:1-O(9S,10R)/17:0)** |  |  |  |  | **1.63** |  |  |  |
| **PC(18:3-OH/P-16:0)** |  | **0.69** |  |  |  |  |  |  |
| **PC(18:3/0:0)** |  |  |  |  |  |  | **1.12** | **1.15** |
| **PC(19:0/0:0)** |  |  | **0.84** |  |  |  | **1.31** |  |
| **PC(20:2/0:0)** |  |  |  |  |  |  |  | **1.12** |
| **PC(20:3/0:0)** |  |  | **0.87** |  |  |  |  | **1.25** |
| **PC(20:4/0:0)** |  |  |  |  |  |  |  | **1.14** |
| **PC(22:5/0:0)** |  |  | **0.85** |  |  |  |  |  |
| **PC(O-1:0/16:0)** |  |  | **0.63** |  |  |  |  |  |
| **PC(O-16:0/0:0)** |  |  | **0.90** |  |  |  | **1.18** |  |
| **PC(O-16:0/1:0)** |  |  | **0.90** |  |  |  |  |  |
| **PC(O-16:0/3:0)** | **0.94** |  |  |  |  |  |  |  |
| **PC(O-16:1/0:0)** |  |  |  |  |  |  | **1.17** |  |
| **PC(O-18:0/0:0)** |  |  | **0.84** |  |  |  | **1.12** |  |
| **PC(O-18:1/0:0)** |  |  |  |  |  |  | **1.23** |  |
| **PC(O-18:1/2:0)** |  |  | **0.85** |  |  |  |  |  |
| **PC(P-14:0/0:0)** |  |  |  |  |  |  | **1.25** |  |
| **PC(P-16:0/0:0)** |  |  | **0.89** |  |  |  |  |  |
| **PC(P-17:0/0:0)** |  |  | **0.87** |  |  |  | **1.20** |  |
| **PC(P-20:0/0:0)** |  |  |  |  |  |  | **1.39** |  |
| PON-PC |  |  |  | 0.54 |  |  |  | 5.09 |
| PS(P-20:0/19:1) |  |  | 0.75 |  |  |  |  |  |
| PS-PC |  |  |  |  |  |  | 1.14 |  |
| sn-glycero-3-phosphocholine |  |  |  |  |  |  |  | 1.39 |

Fold change <1 and >1 indicates uptake and secretion of circulating metabolites by brain, respectively.

Supplementary Table 10. Fold changes of levels of compounds in other subclasses with significant difference between artery and jugular bulb.

| Metabolites | Fold change of metabolite levels | | | | | | | |
| --- | --- | --- | --- | --- | --- | --- | --- | --- |
|  | Group S | | | | Group P | | | |
|  | T0 | T1 | T2 | T3 | T0 | T1 | T2 | T3 |
| (+)-desoxyscalarin |  |  |  |  |  |  | 1.18 |  |
| (+/-)-dihydrofarnesol |  | 2.27 |  |  |  |  |  |  |
| (-)-Malyngolide | 0.86 |  |  |  |  |  |  |  |
| (-)-spongiane-15,16-diol |  |  | 0.68 |  |  |  | 0.56 |  |
| (-)-trans-3,4-dihydro-4,8-dihydroxy-3-methyl-1H-2-benzopyran-1-one |  |  |  |  |  |  | 1.91 | 2.25 |
| (1R,3S,4S,6R)-6,9-dihydroxyfenchone 6-O-b-D-glucoside | 1.50 |  |  |  |  |  | 1.44 |  |
| (2-amino-3-hydroxyoctadecyl) dihydrogen phosphate |  |  |  |  |  | 1.06 |  |  |
| (3β,22R,23R,24S)-3,22,23-trihydroxystigmastan-6-one |  |  |  |  |  |  | 1.17 |  |
| (3a,5b,7a)-23-carboxy-7-hydroxy-24-norcholan-3-yl-b-D-glucopyranosiduronic acid |  |  | 1.79 |  |  |  |  |  |
| (3Z)-phytochromobilin |  |  | 1.39 |  | 0.83 |  |  |  |
| (4R)-2-undecyl-4-thiazolidinecarboxylic acid |  |  | 0.85 |  |  |  |  |  |
| (4R,6S)-4-hydroxy-1,7-dioxaspiro[5.5]undecane |  |  |  |  |  | 0.67 |  |  |
| (22E,24R)-stigmasta-4,22-diene-3,6-dione | 1.16 |  |  |  |  |  |  |  |
| (25R)-5β-spirostan-1β,3α-diol | 1.41 |  |  |  |  |  | 1.35 |  |
| (E)-2-tridecene-4,6,8-triyn-1-ol |  |  | 0.72 |  |  |  |  |  |
| (R)-3-hydroxybutanoic acid |  |  |  |  | 0.87 | 0.90 |  |  |
| (S)-3,4-dihydroxybutyric acid |  |  |  |  |  | 0.93 |  |  |
| [(3S,4S,5S,6R)-3,4,5-trihydroxy-6-(hydroxymethyl)oxan-2-yl] octadec-9-enoate |  |  |  | 1.17 |  |  |  |  |
| 1-(2-hydroxyethyl)-2-hydroxymethyl-5-nitroimidazole |  |  |  |  |  |  |  | 0.87 |
| 1-(2-methoxy-6Z-heptadecenyl)-sn-glycero-3-phosphoethanolamine |  |  | 0.85 |  |  |  |  |  |
| 1-{2-[(3-ethylphenyl)amino]-2-oxoethyl}-6-oxo-1,6-dihydropyridine-3-carboxylic acid |  |  |  |  |  |  |  | 1.47 |
| 1-α,18,25-trihydroxyvitamin D3 |  |  | 1.09 |  |  |  |  |  |
| 1-α,25-dihydroxy-24a,24b-dihomovitamin D3 |  |  |  |  |  | 1.14 |  |  |
| 1-acetylindole |  |  |  |  |  | 0.91 |  |  |
| 1-hydroxy-5-phenyl-3-pentanone |  |  |  | 1.18 |  |  |  |  |
| 1-keto-24-methylcalcifediol |  |  |  |  |  |  | 1.25 |  |
| 1-monolinolenoyl-rac-glycerol | 7.21 | 1.40 |  | 2.56 | 5.20 |  | 2.93 | 2.85 |
| 1-O-(2R-methoxy-4Z,7Z,10Z,13Z,16Z-docosapentaenyl)-sn-glycerol |  |  |  |  |  | 1.24 | 1.26 |  |
| 1-O-(6r-(3,6-dideoxy-α-l-arabinopyranosyloxy)-heptyl)-β-d-glucopyranose |  |  |  |  |  |  | 1.18 |  |
| 1,25-dihydroxy-16-ene-vitamin D3 |  |  |  |  |  |  | 1.25 |  |
| 2-(5,8-tetradecadienyl)cyclobutanone |  |  |  |  |  |  | 2.01 |  |
| 2-(8-[5]-ladderane-octanyl)-sn-glycerol | 1.63 | 1.60 |  | 0.63 |  |  |  |  |
| 2-(p-acetamidophenyl)-2-ethylglutarimide |  |  |  |  |  | 1.22 |  | 1.27 |
| 2-C-methyl-1,4-erythrono-D-lactone | 0.82 |  |  |  |  |  |  |  |
| 2-deoxyglucose |  |  |  | 0.85 |  |  |  |  |
| 2-furoic acid | 0.85 |  |  |  |  |  |  |  |
| 2-hydroxy-decanedioic acid |  |  |  |  |  | 0.63 |  |  |
| 2-methylindoline |  |  |  |  |  | 1.32 |  | 1.32 |
| 2-methyl-4-oxopentanedioic acid |  |  |  |  |  |  |  | 0.85 |
| 2-methyl-4-phenylbutyraldehyde |  |  | 0.73 |  |  |  |  |  |
| 2-oxobutanoic acid |  |  |  |  |  | 0.93 |  |  |
| 2-succinylbenzoate |  |  |  |  |  |  | 0.80 |  |
| 2,3-dihydrobenzofuran |  |  |  |  |  |  | 1.35 |  |
| 2,3,6,7-tetrahydrocyclopent[b]azepin-8(1H)-one |  |  | 1.34 |  |  |  | 1.51 |  |
| 2,6-di-tert-butyl-4-methylene-2,5-cyclohexadienone | 0.87 |  |  |  |  |  |  |  |
| 3'-hydroxyamobarbital |  |  |  | 0.76 |  |  |  |  |
| 3-(1-azatricyclo[7.3.1.05,13]trideca-5,7,9(13)-trien-7-yl)-2-cyanoprop-2-enoic acid |  |  |  | 0.88 |  |  |  | 1.53 |
| 3-(5,6,6-trimethylbicyclo[2.2.1]hept-1-yl)cyclohexanol | 1.61 |  |  |  | 2.04 |  | 1.25 | 1.82 |
| 3-butyrl propionic acid |  |  |  | 1.64 |  |  |  |  |
| 3-hydroxy-dodecanedioic acid | 0.83 |  |  |  |  |  |  |  |
| 3-hydroxyadipic acid 3,6-lactone | 0.76 |  |  |  |  |  |  |  |
| 3-methyl-1-phenyl-3-pentanol |  | 1.31 |  |  |  |  |  |  |
| 3-methyl-2-oxobutanoic acid |  |  |  |  |  |  |  | 0.88 |
| 3-methyldioxyindole |  |  |  |  |  |  | 0.88 |  |
| 3-O-α-L-rhamnopyranosyl-3-hydroxy-5Z-tetradecenoyl-3-hydroxydecanoic acid |  | 1.55 |  |  |  |  |  |  |
| 3-oxo capric acid |  |  |  |  | 1.29 |  |  |  |
| 3,4-dihydro-6-methoxy-2,2-dimethyl-2H-1-benzopyran-4-ol |  |  |  |  |  |  | 1.18 |  |
| 3,6-dimethyl-1,4-dioxane-2,5-dione |  |  |  |  |  |  | 0.88 |  |
| 4-coumaroylshikimate |  |  |  |  |  | 0.74 |  |  |
| 4-hydroxy-4-methyl-5-hexenoic acid gamma lactone |  |  |  |  |  |  | 0.89 |  |
| 4-methyl-2-pentyloxazole | 0.75 |  |  |  |  |  |  |  |
| 4-methyl-3-heptyl linoleate |  |  |  |  |  | 1.21 |  |  |
| 4-O-methylmelleolide |  |  |  |  |  |  | 1.15 |  |
| 4-oxododecanedioic acid | 0.85 |  |  |  |  |  |  |  |
| 5'-carboxy-α-chromanol |  |  |  |  |  |  | 1.22 |  |
| 5-(1-hydroxypropan-2-yl)isolongifol-5-ene |  |  |  |  |  | 1.10 |  |  |
| 5-(11S-hydroxy-8Z-heptadecenyl) resorcinol |  |  |  |  |  |  | 1.14 |  |
| 5-(hexahydro-2-oxo-1H-thieno[3,4-D]imidazol-6-YL)pentanal |  |  |  | 0.78 |  |  |  |  |
| 5-ethyl-3-hydroxy-4-methyl-2(5H)-furanone | 0.82 |  |  |  |  |  |  |  |
| 5-hydroxy-1-methylpyrrolidin-2-one | 0.76 |  |  |  |  |  |  |  |
| 5-keto-6E,8E-octadecadienoic acid |  |  | 1.38 |  |  |  |  |  |
| 5,6-dihydroxyindole |  |  |  |  |  |  |  | 0.41 |
| 5,6-Ep-15S-HETE |  |  | 0.69 |  |  |  | 0.79 |  |
| 5α-cholest-25-ene-3α,7α,12α,26-tetrol |  |  | 1.06 |  |  |  |  |  |
| 5α-cholestan-3α,12α,16α-triol |  |  |  |  |  |  | 1.13 |  |
| 5α-pregnan-20α-ol-3-one |  |  |  |  |  |  | 1.13 |  |
| 6-[(3,4-methylenedioxy)phenyl]-3,3-dimethyl-1-hexene |  |  |  |  |  |  | 1.90 |  |
| 6-[5]-ladderane-1-hexanol | 8.31 |  | 2.70 | 1.54 | 9.23 |  | 5.05 |  |
| 6-α-acetoxy-hopane-16-β,22-diol |  |  |  |  |  |  | 1.61 |  |
| 6-O-acetylaustroinulin |  |  |  |  |  | 1.12 |  |  |
| 7α-hydroxy-4-cholesten-3-one |  |  |  |  |  |  | 1.45 |  |
| 7β-acetoxy-gorgostan-3β,5α,6β-triol |  |  |  |  |  | 1.21 |  |  |
| 7a,17-dimethyl-5b-androstane-3a,17b-diol |  |  |  |  |  |  | 1.13 |  |
| 7E,10-undecadien-4-olide |  |  | 1.15 |  |  |  |  |  |
| 7S,8S-DiHODE | 0.81 |  |  |  |  |  |  |  |
| 9-hydroxylinoleic acid |  |  | 1.21 |  |  |  |  |  |
| 9-hydroxymethyl-10-carbamoylacridan |  |  |  | 1.22 |  |  |  | 1.79 |
| 9R-HODE | 0.77 |  |  |  |  |  |  |  |
| 9R,10S-epoxy-3Z,6Z-eicosadiene | 1.41 |  |  |  |  |  |  |  |
| 10-(phosphonooxy)decyl methacrylate |  |  |  |  |  |  | 1.42 |  |
| 10-hydroxycarbazepine |  |  | 1.16 |  |  |  |  | 1.32 |
| 10-oxo-11-octadecen-13-olide |  |  | 1.18 |  |  |  |  |  |
| 10,12,15-octadecatrienoic acid |  |  |  |  |  | 1.11 |  |  |
| 10E,14E-octadecadienoic acid |  |  |  |  |  | 1.11 |  |  |
| 11-dehydrocorticosterone |  | 0.61 |  | 0.59 | 0.48 | 0.41 |  |  |
| 11α-ethynyl-1α,25-dihydroxyvitamin D3 |  |  | 1.29 |  |  |  |  |  |
| 11β-hydroxyprogesterone |  |  |  | 0.71 |  |  |  |  |
| 11Z,14Z-octadecadienoic acid |  |  |  |  |  | 1.20 |  | 1.22 |
| 12-oxo-14,18-dihydroxy-9Z,13E,15Z-octadecatrienoic acid |  |  |  |  |  |  |  | 1.28 |
| 12β,20S,-trihydroxydammar-3,25-dione |  |  |  | 0.89 |  |  |  |  |
| 13(S)-HODE methyl ester | 9.01 |  | 3.09 |  | 6.48 |  | 2.73 | 3.60 |
| 13-hydroxyoctadeca-9,12-dienoic acid |  |  |  |  | 1.12 |  |  |  |
| 13S-HOTrE |  |  |  |  |  | 1.07 |  |  |
| 15,16-dihydrosacrolide A |  |  |  | 0.78 |  |  |  |  |
| 16-O-deacetyl-16-epi-scalarolbutenolide |  |  |  |  |  |  | 1.11 |  |
| 17-tetracosenal |  |  |  |  | 0.74 | 1.58 |  |  |
| 19-norandrosterone |  |  |  |  |  | 1.17 |  |  |
| 20S-hydroxycholestane-3,16-dione | 1.34 |  |  |  | 1.20 |  |  |  |
| 25-hydroxy-6,19-dihydro-6,19-ethanovitamin D3 | 1.16 |  |  |  |  |  |  |  |
| 25,26-dihydroxyvitamin D3 | 2.04 | 1.48 | 1.84 | 1.65 | 2.07 | 2.22 | 2.19 | 1.79 |
| α-bisabolol oxide A | 0.81 |  |  |  |  |  |  |  |
| α-carboxydimethyldecylhydroxychroman | 1.23 |  |  |  |  |  |  |  |
| α-carboxyethyl hydroxychroman |  |  |  | 1.18 |  |  |  |  |
| β-guanidinopropionic acid |  |  |  |  | 0.62 |  |  |  |
| β-sitostenone | 1.89 |  |  |  |  |  |  |  |
| δ1-nonen-3-ol |  |  | 1.18 |  |  |  |  |  |
| allithiamine |  |  | 1.40 |  |  |  |  | 2.35 |
| annocherin A |  |  |  |  |  |  | 1.13 |  |
| anordiol | 1.34 |  |  |  |  |  |  |  |
| arachidonoyl amine |  |  |  | 0.52 |  |  |  |  |
| aragusteroketal |  |  |  |  |  |  | 1.14 |  |
| ascladiol |  |  |  | 1.12 |  |  | 0.85 |  |
| auberganol |  |  |  | 1.08 |  |  |  |  |
| avocadene 4-acetate |  |  |  |  |  | 1.20 | 1.13 |  |
| avocadenyne acetate |  |  | 1.61 |  |  |  | 1.95 |  |
| azelnidipine |  |  |  | 1.13 |  |  |  |  |
| Bacchabolivic acid |  |  |  |  |  |  | 1.29 |  |
| bilirubin |  |  | 3.13 |  |  |  |  | 0.09 |
| bisacumol | 0.83 |  |  |  |  |  |  |  |
| butyl (S)-3-hydroxybutyrate [arabinosyl-(1->6)-glucoside] |  |  | 7.67 |  |  |  |  | 4.95 |
| campesteryl glucoside |  |  |  |  |  | 1.26 | 1.44 |  |
| carbestrol | 1.24 |  |  |  |  |  |  |  |
| carnosic acid |  |  |  |  |  |  | 1.15 |  |
| cefazolin |  |  |  | 0.80 |  |  |  |  |
| **choline** |  |  |  |  |  | **1.30** | **1.09** |  |
| clausarinol |  |  |  |  |  |  | 1.19 |  |
| conicasterol D |  |  |  |  |  |  | 1.14 |  |
| cucujolide II |  |  |  |  |  | 0.84 |  |  |
| curcolonol | 1.29 |  |  |  |  |  |  |  |
| cyclandelate |  |  |  |  | 1.09 |  |  |  |
| **D-lactic acid** |  |  | **0.76** |  | **0.78** |  | **0.78** |  |
| DG(i-12:0/13:0/0:0) |  |  |  |  |  |  | 0.84 |  |
| DG(i-13:0/10:0/0:0) | 1.27 |  |  |  |  |  |  |  |
| DG(i-13:0/18:1-O(9S,10R)/0:0) | 1.25 |  |  | 0.92 |  |  |  |  |
| DG(i-13:0/20:4-OH/0:0) |  |  |  |  | 1.33 |  |  |  |
| DG(2:0/0:0/18:3-OH) |  |  | 1.23 |  |  |  |  |  |
| DG(13:0/18:1-O(9S,10R)/0:0) |  |  |  | 0.82 |  | 1.16 |  |  |
| digalogenin |  |  |  |  |  | 1.14 |  |  |
| dihydromethysticin |  |  |  | 1.24 |  | 2.83 |  |  |
| dimethylmalonic acid | 0.82 |  |  |  |  |  |  |  |
| doxercalciferol | 1.46 |  |  | 0.73 |  |  |  |  |
| Durandro | 1.73 | 1.41 |  |  |  |  |  | 1.36 |
| emmotin A | 0.52 | 0.67 |  |  |  |  |  |  |
| enigmol |  |  | 0.82 |  |  | 1.14 | 1.27 |  |
| ent-6,16-kauradien-19-oic acid | 1.24 |  |  |  | 1.23 |  |  |  |
| eremopetasidione | 0.85 |  |  | 1.12 |  |  |  |  |
| eremopetasinorol |  |  | 1.29 |  |  |  |  |  |
| erinapyrone A |  |  |  |  |  | 0.85 |  |  |
| erythroskyrin |  | 1.46 |  |  |  |  | 2.80 |  |
| estrane-3α,17α-diol | 0.87 |  |  |  |  |  |  |  |
| etretinate | 0.83 |  |  |  |  |  |  |  |
| fluvoxamino acid |  |  | 1.45 |  |  |  |  |  |
| fosteabine |  |  |  |  |  |  | 1.16 |  |
| furanofukinin | 0.93 |  |  |  |  |  |  | 0.95 |
| fusarochromanone |  |  |  |  |  |  |  | 4.11 |
| galaxaurol D |  |  |  |  |  |  | 1.14 |  |
| gallein |  |  |  |  |  | 2.78 |  |  |
| ganoderal A |  |  |  |  |  |  |  | 2.12 |
| gentioflavine | 0.72 |  |  |  |  |  |  |  |
| gibberellin A86 |  |  | 0.78 |  |  |  |  |  |
| glutaconic acid |  |  | 0.74 |  |  |  |  |  |
| glycochenodeoxycholic acid 3-glucuronide |  |  |  |  |  | 1.20 |  |  |
| heptaethylene glycol |  |  | 0.85 |  |  |  |  |  |
| homobutein 4'-O-glucoside | 2.17 |  |  |  |  |  |  |  |
| homoveratric acid |  | 0.86 |  |  |  |  |  |  |
| humulol |  |  | 1.23 |  |  |  |  |  |
| hypoxanthine | 0.87 |  | 0.86 |  |  |  |  |  |
| ieodomycin A |  |  |  | 1.21 |  |  |  |  |
| ieodomycin B |  |  |  |  |  | 0.72 |  |  |
| indolepyruvate |  |  |  |  |  |  |  | 2.13 |
| isoalantolactone |  |  |  |  |  |  | 0.78 |  |
| **isocitric acid** |  |  |  |  |  |  | **1.58** |  |
| janthitrem C |  |  |  |  |  | 1.04 |  | 1.17 |
| ketone Ester |  |  |  |  |  |  | 1.14 |  |
| kuguacin C |  |  |  |  |  |  | 1.15 |  |
| laurelliptine |  |  |  | 0.80 |  |  |  |  |
| levonorgestrel acetate |  |  |  | 0.47 |  |  |  |  |
| lufenuron |  |  |  |  | 0.72 |  |  |  |
| luprostiol |  |  |  |  |  | 2.55 |  |  |
| lycocasuarinen acid C |  |  |  |  |  |  | 0.93 |  |
| Macaene |  |  |  |  |  |  |  | 1.15 |
| malic acid |  | 0.75 |  |  |  |  |  |  |
| methyl 6,7-dimethoxy-4-ethyl-β-carboline-3-carboxylate |  |  |  |  |  |  |  | 1.33 |
| methyl jasmonate | 0.75 |  |  |  |  |  |  |  |
| methyl-[12]-gingerdiol |  |  |  |  |  | 1.33 |  |  |
| methyl-[12]-gingerol |  |  |  |  |  | 1.14 |  |  |
| MG(0:0/18:1/0:0) | 2.07 |  |  |  | 2.61 |  |  | 2.02 |
| MG(0:0/20:3-2OH/0:0) |  | 1.48 |  |  |  |  |  |  |
| MG(0:0/22:1/0:0) |  |  |  | 0.92 |  |  |  |  |
| MG(0:0/22:5/0:0) | 10.8 | 1.95 |  | 4.73 | 9.43 | 1.82 | 4.20 | 4.30 |
| MG(0:0/24:6/0:0) |  |  |  |  |  | 1.26 | 1.25 |  |
| MG(16:0/0:0/0:0)[rac] | 10.7 | 1.81 |  |  | 5.17 |  |  | 3.66 |
| MG(17:0/0:0/0:0)[rac] |  |  |  |  |  |  |  | 1.16 |
| MG(18:1/0:0/0:0) | 4.12 |  |  | 2.23 | 5.70 |  | 2.57 |  |
| MG(18:2/0:0/0:0) | 3.57 |  |  |  | 2.82 |  | 2.19 | 2.03 |
| MG(18:2/0:0/0:0)[rac] | 4.20 |  |  |  | 4.64 |  | 2.52 |  |
| MG(20:4/0:0/0:0) | 7.06 | 1.55 |  | 2.42 | 4.59 |  | 4.22 | 3.09 |
| MG(22:5/0:0/0:0) | 4.31 | 1.53 | 1.68 |  | 4.07 |  | 2.13 |  |
| MG(22:6/0:0/0:0) | 10.7 | 1.97 |  | 3.91 | 7.87 |  | 5.19 | 4.03 |
| MGMG(16:2/0:0) |  |  |  |  | 1.45 |  |  |  |
| modiolide A |  |  | 1.05 |  |  | 0.90 |  |  |
| N-(6-aminohexyl)-5-chloro-1-naphthalenesulfonamide |  |  | 1.25 |  |  |  |  |  |
| N-desmethyl-p-hydroxyrosiglitazone |  |  |  |  |  |  | 0.55 |  |
| nandrolone |  | 1.41 |  |  |  |  |  | 1.91 |
| nepetaside |  |  |  |  |  |  | 1.40 |  |
| norambreinolide |  | 1.83 |  |  | 1.07 | 1.08 |  |  |
| oxandrolone | 1.33 |  |  |  |  |  |  |  |
| oxendolone |  |  |  |  |  | 1.24 |  |  |
| p-hydroxycinnamic acid |  |  | 1.62 | 1.66 |  |  |  |  |
| panaquinquecol 2 | 1.24 |  |  |  |  |  |  |  |
| paramethadione |  |  |  |  | 1.19 |  | 1.26 |  |
| paricalcitol |  |  |  |  |  |  | 1.12 |  |
| PC(20:4-OH(18R)/P-16:0) |  |  |  |  |  | 0.78 |  |  |
| **PE(P-16:0/0:0)** |  |  | **0.92** |  |  |  |  | **1.14** |
| **PE(0:0/16:0)** |  |  |  |  |  |  | **1.10** |  |
| **PE(0:0/18:3)** | **1.23** |  |  | **0.81** |  |  |  |  |
| **PE(0:0/20:1)** |  |  |  | **0.81** |  |  |  |  |
| **PE(0:0/20:2)** |  |  |  | **0.79** |  |  | **1.17** |  |
| **PE(0:0/20:4)** |  |  |  | **0.93** |  |  |  |  |
| **PE(0:0/22:6)** |  |  |  |  |  |  | **1.12** |  |
| **PE(18:1/0:0)** |  |  |  | **0.83** |  |  |  |  |
| **PE(20:2/19:0)** |  |  |  |  |  | **0.94** |  |  |
| **PE(20:3/0:0)** |  |  |  | **0.86** |  |  |  |  |
| **PE(20:4/0:0)** |  |  |  | **0.77** |  |  |  |  |
| **PE(22:0/0:0)** |  |  |  |  |  |  | **1.19** |  |
| **PE(22:1/0:0)** |  |  | **0.51** |  |  |  |  |  |
| **PE(22:4/0:0)** | **1.18** |  |  |  |  |  |  |  |
| **PE(O-18:0/0:0)** |  |  |  |  |  | **1.12** |  |  |
| persenone B |  |  |  |  |  |  |  | 1.40 |
| phenol A |  |  | 1.18 |  |  |  |  |  |
| PI(12:0/0:0) |  |  |  | 1.82 |  |  |  |  |
| PI(20:0/18:2+=O) |  |  |  |  |  |  | 0.69 |  |
| pimonidazole | 1.62 |  |  |  |  |  | 1.54 |  |
| piromelatine |  |  |  |  |  |  | 0.76 |  |
| polyglycerol esters of fatty acids | 27.5 | 21.9 | 19.8 | 18.5 | 24.4 | 25.9 | 22.4 | 22.4 |
| poststerone |  |  |  |  | 0.76 |  |  |  |
| provitamin D6 |  |  | 1.57 |  |  |  |  |  |
| PS(20:5/0:0) | 1.26 |  |  | 0.73 |  |  |  |  |
| PS(21:0/18:4) |  |  |  |  |  |  |  | 0.76 |
| quadrone |  |  | 0.86 |  |  |  |  |  |
| quercetin 7,3',4'-trimethyl ether 3-rhamnoside |  |  |  | 1.63 |  |  |  |  |
| Reichstein's substance E |  |  |  |  |  |  | 0.64 |  |
| rockogenin | 1.41 |  |  |  |  |  |  |  |
| Rosterolone |  |  |  |  |  |  | 1.15 |  |
| salaspermic acid |  |  |  |  |  |  | 2.03 |  |
| schleicherastatin 5 |  |  |  |  |  |  | 1.21 |  |
| siderol |  | 1.56 |  |  |  |  |  |  |
| sinapyl alcohol |  |  | 1.30 |  |  |  |  |  |
| SM(d18:1/16:0) | 0.76 |  |  |  |  |  | 0.79 |  |
| sphinganine-phosphate |  |  |  |  |  | 1.12 |  |  |
| sphingosine-1-phosphate |  |  | 0.88 |  |  | 1.08 |  |  |
| stearidonic acid | 2.26 |  |  |  |  |  |  | 1.36 |
| Taccagenin |  |  |  |  |  |  | 1.33 |  |
| taxa-4(20),11(12)-dien-5alpha-acetoxy-10beta-ol |  |  |  |  |  | 1.19 |  |  |
| testosterone sulfate |  |  |  |  |  |  | 1.22 |  |
| tetrahydrocortisol |  | 1.33 |  |  |  |  |  |  |
| TG(10:0/8:0/8:0) | 1.48 |  |  |  |  |  |  |  |
| toxin T2 tetrol |  |  |  |  |  |  | 1.62 |  |
| trimethylamine N-oxide |  |  |  |  |  |  |  | 1.37 |
| tromethamine |  |  |  | 2.57 |  |  |  |  |
| tumonoic acid H |  |  | 0.87 |  |  |  |  |  |
| ulinastatin | 0.77 |  |  |  |  |  |  |  |
| urocortisone |  | 1.63 |  |  | 1.38 | 1.38 |  |  |
| viscumneoside VI |  |  |  |  |  | 5.62 |  |  |
| voxtalisib |  |  |  |  |  |  |  | 2.17 |
| xeniasterol-a |  |  |  |  |  | 1.25 | 1.31 |  |
| xeniasterol-b |  |  |  |  |  | 1.11 | 1.22 |  |

Fold change <1 and >1 indicates uptake and secretion of circulating metabolites by brain, respectively.
